# Trends in Antibiotic Prescribing in Out-of-Hours Primary Care in England from January 2016 to June 2020 to Understand Behaviours during the First Wave of COVID-19

**DOI:** 10.3390/antibiotics10010032

**Published:** 2021-01-01

**Authors:** Nina J. Zhu, Monsey McLeod, Cliodna A. M. McNulty, Donna M. Lecky, Alison H. Holmes, Raheelah Ahmad

**Affiliations:** 1 National Institute for Health Research, Health Protection Research Unit in Healthcare Associated Infections and Antimicrobial Resistance, Imperial College London, London W12 0NN, UK; jiayue.zhu09@imperial.ac.uk (N.J.Z.); monsey.mcleod@nhs.net (M.M.); alison.holmes@imperial.ac.uk (A.H.H.); 2 Centre for Medication Safety and Service Quality, Pharmacy Department, Imperial College Healthcare NHS Trust, London W12 0NN, UK; 3 National Institute for Health Research, Imperial Patient Safety Translational Research Centre, Imperial College London, London W2 1PG, UK; 4 Primary Care Unit, Public Health England, Gloucestershire GL1 1DQ, UK; cliodna.mcnulty@phe.gov.uk (C.A.M.M.); donna.lecky@phe.gov.uk (D.M.L.); 5 Centre for Antimicrobial Optimisation, Imperial College London, London W12 0NN, UK; 6 Department of Infectious Diseases, Imperial College London, London SW7 2AZ, UK; 7 Imperial College Healthcare NHS Trust, Hammersmith Hospital, London W12 0NN, UK; 8 Division of Health Services Research and Management, School of Health Sciences, University of London, London EC1V 0HB, UK

**Keywords:** out-of-hours, antibiotics, antimicrobial stewardship, COVID-19

## Abstract

We describe the trend of antibiotic prescribing in out-of-hours (OOH) general practices (GP) before and during England’s first wave of the COVID-19 pandemic. We analysed practice-level prescribing records between January 2016 to June 2020 to report the trends for the total prescribing volume, prescribing of broad-spectrum antibiotics and key agents included in the national Quality Premium. We performed a time-series analysis to detect measurable changes in the prescribing volume associated with COVID-19. Before COVID-19, the total prescribing volume and the percentage of broad-spectrum antibiotics continued to decrease in-hours (IH). The prescribing of broad-spectrum antibiotics was higher in OOH (OOH: 10.1%, IH: 8.7%), but a consistent decrease in the trimethoprim-to-nitrofurantoin ratio was observed OOH. The OOH antibiotic prescribing volume diverged from the historical trend in March 2020 and started to decrease by 5088 items per month. Broad-spectrum antibiotic prescribing started to increase in OOH and IH. In OOH, co-amoxiclav and doxycycline peaked in March to May in 2020, which was out of sync with seasonality peaks (Winter) in previous years. While this increase might be explained by the implementation of the national guideline to use co-amoxiclav and doxycycline to manage pneumonia in the community during COVID-19, further investigation is required to see whether the observed reduction in OOH antibiotic prescribing persists and how this reduction might influence antimicrobial resistance and patient outcomes.

## 1. Introduction

Antimicrobial Resistance (AMR), the loss of effectiveness of anti-infective (antiviral, antifungal, antibacterial and antiparasitic) medicines, jeopardises our ability to treat infections [1]. Progress against the UK 5-year action plan for AMR continues to highlight that the inappropriate use of antimicrobials in human medicine is one of the main drivers of AMR in the UK and internationally, with at least 20% of the antibiotics prescribed in UK primary care being inappropriate [2]. The real figure may be much higher due to poor diagnostic coding in primary care [3,4]. In England, primary care settings include General Practice (GP) and *other community settings*. GP practice prescribing during routine hours accounts for about 85% of primary care antibiotic items prescribed [5]. Other community settings include: out-of-hours (OOH) services, walk-in centres, urgent & emergency care, community health service, optometry services, hospices, care homes, nursing homes, and custody [5]. Among these other community settings, the highest prescribing rate occurs in OOH services, which account for 53.6% of prescribing within this category and 3.3% of prescribing in primary care [6]. In England, OOH services run from 6.30 p.m. to 8.00 a.m. on weekdays and all day on weekends and on bank holidays to provide treatment and advice for patients whose medical issues are not life-threatening but urgent, when GP surgeries are typically closed [7]. A range of antimicrobial stewardship, educational and policy interventions have been implemented in England, aimed at improving the quality of antibiotic prescribing [8]. One such initiative, the antibiotic Quality Premium, a financial incentive for local commissioners for targeted reductions in primary care antibiotic prescribing, was introduced in England in April 2015 with a focus on urinary tract infection management and a reduction in co-amoxiclav and quinolones [9]. Antibiotic consumption data shows that in-hour (IH) GPs have achieved a consistent reduction in antibiotic prescribing since the introduction of the QPs, demonstrating a positive contributing impact of this initiative [10,11]. Despite the improvement in IH GP settings, little has been achieved to improve antibiotic use in OOHs [5]. Existing evidence suggests that the antibiotic prescribing volume is higher in OOH services than in IH GP practices for the same patient population. Nationwide, the proportion of prescriptions of broad-spectrum agents decreased in OOH and IH between 2010 and 2014; however, this proportion was always higher in OOH compared with IH [12]. There are regional variations; for example, a study in Oxfordshire shows a trend of an increased number of OOH antibiotic prescriptions between 2010 and 2014 against a decreased number of consultations [13,14]. Limited information is available to clarify whether GP prescribing has been displaced from in-hour general practices to other settings including OOH services.

There is little research exploring OOH antibiotic prescribing and limited AMS interventions specifically tailored to this setting [15]. However, assessing antibiotic use in OOH has become increasingly important given that the utilisation of OOH services has increased year on year and in relation to hospital Acute & Emergency (A&E) centres since 2017 [16]. The ongoing acute viral pandemic caused by SARS-CoV-2 (COVID-19) has driven a rapid reconfiguration of primary care provision in England. Patients were re-directed away from acute hospital and OOHs healthcare facilities to reduce the infection transmission and ‘protect the NHS’; this was done either by healthcare triage or voluntarily by patients [17,18]. Lockdown and the need for social and physical distancing have led to an increase in remote GP consultations. Data collected from more than 500 GP practices in England by the Royal College of General Practice shows that during the first wave of the pandemic (April 2020), around 71% of GP consultations were conducted remotely by telephone or video and 25% were conducted face to face. For the same period in 2019, 25% of consultations were conducted remotely and 70% face to face [19,20]. Remote consultation is reported to have increased clinical uncertainty in infection management without the benefit of an in-person assessment and diagnostic tests IH and OOH [21]. This is in addition to the challenges already faced by OOH GP staff (being unfamiliar with patients) and usually without access to the patient’s complete medical history [22]. The OOH setting is also diverse in the range of providers and types of contracts and teams involved. During the COVID-19 pandemic prevaccination, OOH practices and their staff were used to support urgent care centres and walk-in centres designated to provide face-to-face consultations for respiratory patients; referred to as the ‘hot hubs’, these practices or medical centres acted as sites where patients with coronavirus symptoms were seen face to face [17]. 

The aim of this study is to investigate any changes in OOH antibiotic use prior to and during England’s first wave of the COVID-19 pandemic. 

## 2. Results

### 2.1. Antibiotic Prescribing in GP In-Hours and Out-of-Hours

Antibiotic prescribing in OOH settings measured in terms of antibiotic items represented 3.3–3.6% of the total primary care antibiotic prescriptions from 2016 to 2019. (Table 1).

**Figure 1:**
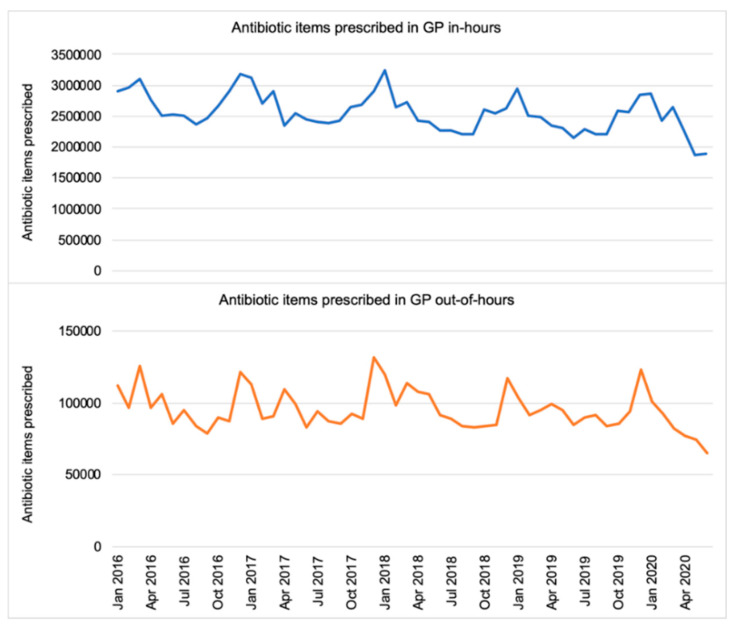
Total number of antibiotic items prescribed (England, 2016–2020).

The prescription volume peaked each year in December for both IH and OOH GP (Figure 1). Prior to the COVID-19 pandemic (from January 2016 to February 2020), IH GP prescribing followed a downward trend; a decrease of 7329 items per month (95% CI: −12,478.14 to −2179.61, *p* < 0.05). For the same period, there was no statistically significant change in the number of items prescribed monthly in OOH (*p* = 0.42). Measured by the number of items, penicillins remained the most commonly prescribed antibiotic group (61.6%) by OOH GPs, followed by tetracyclines (11.2%) and macrolides (10.30%), which were the same as for IH GPs. Each year, the proportion of broad-spectrum antibiotics (cephalosporins, quinolones and co-amoxiclav) to the total prescription volume was the highest in August, both among IH and OOH GPs. 

**Figure 2:**
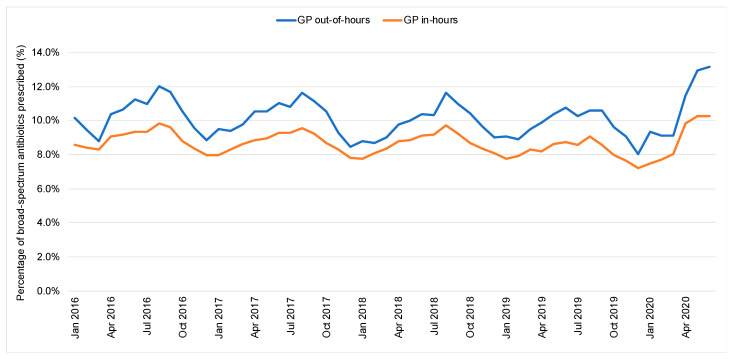
Percentage of broad-spectrum antibiotics prescribed (England, 2016–2020).

The proportion of broad-spectrum antibiotics to total antibiotic prescribing in OOH was consistently higher (10.1%, 95% CI: 10.0% to 10.2%, *p* < 0.05) compared with in-hours (8.7%, 95% CI: 8.6% to 8.8%, *p* < 0.05) (Figure 2). Before March 2020, the proportion of prescribed broad-spectrum antibiotics decreasing by 0.02% per month in GP IH settings (95% CI: −0.03% to −0.01%, *p* < 0.05). While a decrease of 0.02% per month was observed in the proportion of prescribed broad-spectrum antibiotics in OOH, this is insignificant (*p* = 0.06).

### 2.2. Respiratory Tract and Chest Infection Antibiotics in Out-of-Hours

**Figure 3:**
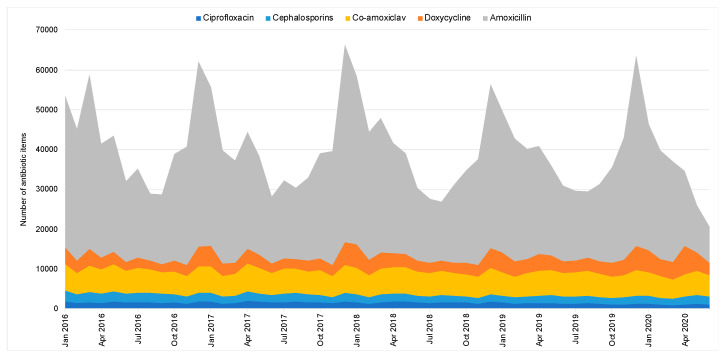
Respiratory tract and chest infection antibiotic items prescribed in out-of-hours (England, 2016–2020).

While the OOH prescribing of ciprofloxacin and cephalosporins remained stable within each year, the prescribing of co-amoxiclav, amoxicillin and doxycycline peaked in December every year, with the seasonality indicating a high use of amoxicillin in winter to treat respiratory tract infections (Figure 3). In 2020, the OOH use of co-amoxiclav and doxycycline peaked between March to May, which was out of sync with the seasonality peaks (Winter) in previous years (Figure 3).

### 2.3. Urinary Tract Infection Antibiotics in Out-of-Hours

**Figure 4:**
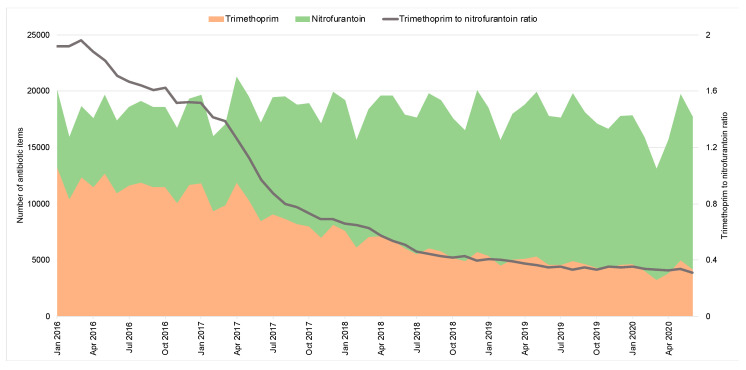
Urinary tract infection antibiotic items prescribed in out-of-hours (England, 2016–2020).

Between 2016 and 2020, there has been a consistent reduction in trimethoprim prescribing and the trimethoprim-to nitrofurantoin-ratio in OOH settings (Figure 4) from 1.9 in January 2016 to 0.3 in June 2020. 

### 2.4. Out-of-Hours Antibiotic Prescribing in the Context of the COVID-19 Pandemic

**Figure 5:**
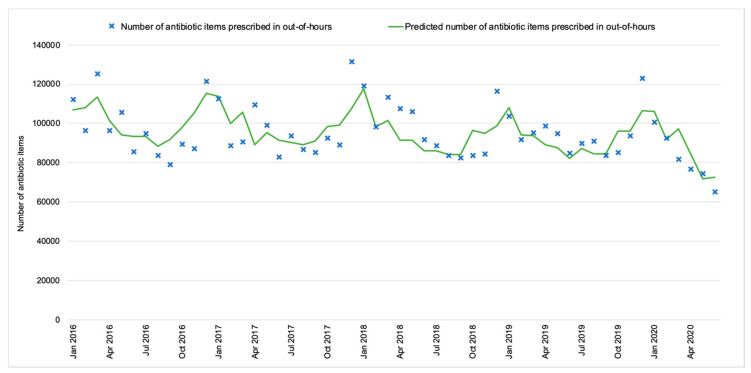
Out-of-hours antibiotic prescribing against the predicted trend (England, 2016–2020).

The trend of the OOH antibiotic prescribing volume between January 2016 and February 2020 remained stable. Since March 2020, there has been a decrease of 5088 items per month (95% CI: −6264.4 to −3912.0). The predicted monthly OOH antibiotic prescribing volume is presented in Figure 5 to illustrate the variation from the historical trend that would have been expected in the absence of the COVID-19 pandemic (Figure 5).

Since March 2020, the proportion of broad-spectrum prescriptions rose in both settings. In IH, the downward trend of the proportion of broad-spectrum prescribing switched direction and started to rise from March, increasing by 0.7% per month (95% CI: 0.3% to 1.2%, *p* < 0.05). In OOHs, though the proportion of broad-spectrum antibiotic prescribing showed no significant overall change prior to March 2020, it started increasing by 1.4% per month (95% CI: 0.9% to 1.9%, *p* < 0.05) since.

## 3. Discussion

The introduction of national antimicrobial stewardship initiatives has reduced antibiotic prescribing primary care in England. Despite the overall decline in primary care antibiotic prescribing between January 2016 to February 2020, the prescribing volume in OOH remained unchanged. The prescribing of broad-spectrum antibiotics in OOH has been consistently higher than IH, as previously reported using data between 2010 and 2014 from 143 out of 221 Clinical Commissioning Groups (CCGs) in England [12]. The positive impact of the QP that was aimed at increasing the use of nitrofurantoin as the first-line treatment for uncomplicated UTI is evident [23], with a decrease of the trimethoprim-to-nitrofurantoin ratio across primary care including OOH settings, though data for assessing QP in OOH is incomplete. The COVID-19 pandemic and the national response have led to a reduction in prescribing in both IH and OOH GPs since March 2020, which may tell us more about patient health-seeking behaviour than clinician prescribing behaviours. The proportion of broad-spectrum antibiotics from the total prescribed antibiotics has, however, increased in both IH and OOH GPs. Co-amoxiclav and doxycycline use in OOH peaked between March and May 2020, probably due to the uptake and implementation of National Institute for Health and Care Excellence (NICE) prescribing guidelines advising doxycycline for the management of community-acquired pneumonia when the cause was unclear during the COVID-19 pandemic [24]. As a percentage of the total, co-amoxiclav may have increased, as OOH clinicians prescribed broad-spectrum antibiotics more frequently to cover a greater range of potential infections when they were unable to assess their patients face to face. 

We cannot rule out a changing epidemiological profile of infectious diseases due to COVID-19 as a possible factor in the decline in OOH antibiotic prescribing, resulting from control measures and a consequent reduced transmission of other respiratory tract infections. Indeed, PHE’s OOH GP syndromic surveillance data indicates that although on average 23% of all OOH GP contacts in England between 2009 and 2019 were due to acute respiratory infection [25], OOH GP contacts for acute respiratory infections, including sore throat, were lower than expected in 2020. During March to August 2020, the daily OOH contacts due to acute respiratory infection fell to 40% of the baseline based on 10-year historical data [25]. 

Our study has two limitations. The first limitation is associated with the quality of the publicly available data. Not all of the prescribing centres in the NHS BSA prescribing data can be identified in the NHS Digital classification of organisations. Therefore, we had to exclude those prescribing records where the setting could not be assigned. This accounted for 0.04% (10,649/23,975,061) of the total prescribing records. Second, our analysis did not capture how OOH consultations were delivered, especially during COVID-19, when an increase in remote consultations was observed in IH GPs [19,20]. Research in how remote consultations might have influenced antibiotic prescribing in the community is urgently needed [26]. 

Despite the limitations, this study provides the first critical insight into antibiotic prescribing in OOH before and during the COVID-19 pandemic and provides the scope for future research. The ongoing monitoring of consultations, antibiotic prescribing and AMR resistance is required to detect any unintended consequences of the change in antibiotic prescribing in OOHs seen in this study. For instance, the increase in co-amoxiclav use in OOH might lead to a rise in the incidence of Clostridium difficile infections in the community. In the current landscape, we also need to investigate OOH service provisions to better understand the factors influencing prescribing decision-making in OOH. As of 2013, the responsibility for commissioning OOH services in England was delegated to CCGs, which are independent statutory bodies governed by GPs to buy services for the local community from providers including hospitals, social enterprises, voluntary organisations and private firms [27]. The contractual arrangements of OOH services are complex and have undergone significant policy changes in recent years due to factors such as CCG mergers, joint commissioning, and the integration of patient triage, ambulance dispatches and OOH care provision [28]. The periodic change of OOH service providers, regional use of paper-based prescriptions and unintegrated electronic patient record systems have challenged the investigation of OOH antibiotic prescribing [12]. An in-depth analysis is required to assess the variation in the OOH care accessibility and quality between different types of service providers, and to evaluate the existing antimicrobial stewardship policies used by them. Monitoring should continue during and beyond the COVID-19 pandemic to determine the long-term impact of possible clinician prescribing behaviour changes versus patient OOH consulting behaviour changes. 

## 4. Materials and Methods 

### 4.1. Ethical Approval

This study did not require ethical approval as it employed publicly available data. 

### 4.2. Data

Out-of-hours antibiotic prescribing data: We extracted records of electronic-based prescriptions of each prescribing centre in England between January 2016 and June 2020 from the NHS Business Service Authority (BSA) [29,30]. The prescribing data contains the number of prescribed antibiotic items each month by prescribing centre. We used the NHS Digital classification of organisations to identify the type of setting for each prescribing centre [31]. Prescribing centres coded as “OOH” and “OOH + walk-in centres (WIC)” were considered OOH practices. We included prescriptions in the British National Formulary (BNF) Sub-chapter 5.1 (antibacterial drugs) [32]. 

Population data: We extracted the mid-year estimates of the population in each region and CCG from the Office for National Statistics (ONS) for the years 2016 to 2019 [33]. 

### 4.3. Descriptive and Statistical Analysis

We report the trend in the OOH antibiotic prescribing volume in England from January 2016 to June 2020. Using the number of items as the measure of the prescribing volume, the trend is compared with IH GP antibiotic prescribing. We examined key agents in the antibiotic QP primary care component, including nitrofurantoin, trimethoprim, amoxicillin, co-amoxiclav (amoxicillin and clavulanic acid), ciprofloxacin and cephalosporins [34]. In addition, we also assessed the prescribing volume of doxycycline, which is an antibiotic agent recommended for the management of suspected or confirmed pneumonia in adults in the community during COVID-19 [24]. The percentage of broad-spectrum to total antibiotic prescriptions per month during IHs and OOHs was compared using a linear regression. To determine whether there was a measurable change in the underlying trend of the OOH antibiotic prescribing volume before and after the national COVID-19 response was imposed in England on 26 March 2020, we performed an interrupted time series analysis (ITSA). ITSA is a quasi-experimental research design commonly used for the evaluation of longitudinal effects of interventions or incidents that might affect population-level health outcomes [35]. ITSA is particularly useful for assessing outcomes before and after an intervention is introduced when it is not possible or ethical to conduct clinical trials [35]. In this study, we define the intervention as the combination of (a) the national lockdown limiting public movement, (b) the local reconfiguration of primary care provisions including walk-in centre closure or the referral of cases to respiratory hot hubs, and (c) the switch to telephone- and video-based consultations in place of face-to-face interactions. The date of the national lockdown (26 March 2020) was used as the implementation date of the “intervention” [36]. We constructed monthly time series of the number of antibiotic items prescribed in OOH settings for the period from January 2016 to June 2020. We performed ITSA to produce Newey—West standard errors for coefficients estimated by ordinary least squared (OLS) regression, which assumed that the errors followed a first-order autoregressive process [37]. We adjusted for seasonality by including each calendar month as an independent variable in the model [36]. 

All descriptive and statistical analyses were performed in STATA 15 (StataCorp, TX, USA).

## 5. Conclusions

This study has provided an insight into the impact of COVID-19 on antibiotic prescribing in OOH settings in the UK. Further investigation is required to see whether the observed reduction in OOH antibiotic prescribing persists and how this reduction might influence AMR and patient outcomes. Monitoring OOH prescribing requires the same primacy as IH prescribing in order to develop antimicrobial stewardship programmes with relevant underpinning evidence. This is all the more important since, in particular, the boundaries between IH and OOH may have different meanings for the healthcare user in the current and continuing context of COVID-19. 

## Figures and Tables

**Table 1:** Antibiotic items prescribed in different settings.

Year	Prescribed Antibiotic Items			Prescribed Antibiotic Items per 1000 Inhabitants per day			Proportion of Primary Care Volume Prescribed in OOH (%)
	Primary Care	GP In-Hours	GP Out-of-Hours	Primary Care	GP In-Hours	GP Out-of-Hours	
2016	34,881,080	32,844,420	1,178,023	1.7	1.6	0.1	3.4%
2017	33,654,010	31,558,849	1,163,962	1.7	1.6	0.1	3.5%
2018	32,362,944	30,159,393	1,177,075	1.6	1.5	0.1	3.6%
2019	31,714,925	29,429,925	1,136,791	1.5	1.4	0.1	3.6%
2020 ^1,2^	14,855,823	13,933,878	492,244	1.5	1.3	0.1	3.3%

1. Only includes data from January to June 2020; 2. Mid-year population estimate of 2020 was not available when this study was conducted; therefore, the 2019 estimate was used.

## Data Availability

The data presented in this study are available in public domains. All data sources have been referenced in the article.
